# Anæsthesia, Local and General

**Published:** 1896-06

**Authors:** A. C. Hewitt

**Affiliations:** Chicago, Ill.


THE
AMERICAN JOURNAL
—OF—
DENTAL SCIENCE.

VOL. XXX. BALTIMORE, JUNE, 1896. No. 2.

ARTICLE I.

### ANÆSTHESIA, LOCAL AND GENERAL.

BY A. C. HEWITT, M. D., CHICAGO, ILL.

Read before the Iowa State Dental Society.

There comes but seldom in the life of a man so distinguished an honor as you, through your committee, have bestowed upon me. That I, a non-resident of your State, not a member of your society, at such an epoch of your State's profession, should be selected to lead in the discussion of anesthetics, general and local, calls for gratitude that I am unable to express, and yet creates anxieties that I have been unable to suppress.

Though not now a dweller on the soil of Iowa, I know the timber of its people; I know the lines along which your emigrants have come; I know the schools in which Iowa's children are taught. In your State are scholars, statesmen, orators, scientists that rank with the ripest and best of any other.

I know that members of the dental profession of Iowa, who have thus honored me, come fresh from contact with those eminent in their several spheres; themselves quick of apprehension, educated, profound thinkers. What wonder then that upon the opening of this question I grow anxious as I take in the broadening field; the narrowest border of which I can only enter upon. However, remembering the marked kindness with which on former occasions you have received me, and upborne by an increasing confidence in the justness and safety of the views I hold, I will go to the heart of my subject, advancing little of mere theory; striving rather for the practical, that you may be assisted in your labors and that your patrons may receive at your hands the benison of your kindly striving.

It might be more pleasing to you, as it was fascinating to myself, to give the history of the use of anesthetics early ventured upon; to note the dawning of the light then breaking; to moralize upon the desire for fame on one hand, and for money as well; to argue for or against the propriety from a professional standpoint of Drs. Morton and Jackson obtaining a patent for etherization in surgery as they did, making oath to application October 27, 1846; to trace Dr. Crawford W. Long in his country practice in Georgia, no doubt wishing for surgery after giving J. M. Venable ether on March 30, 1842, and painlessly removing a glandular tumor from his neck; to weave fiction with fact in the history of Drs. Long, Jackson, Wells, Morton, Simpson, Colton and Riggs. One, Dr. Wells, having committed suicide; another, Dr. Morton, leaving his sick-bed in a state of wild excitement and dying a few hours later in Central Park, New York; a third, Dr. Jackson, becoming deranged, dying in an asylum; and the fourth, Dr. Long, becoming stricken with paralysis while visiting a patient (Dr. Laird W. Nevius in preface to the "Dictionary of Modern Anesthesia"), but the occasion and time forbid.

### DEFINITIONS.

In order that there may be no misapprehension of premise and conclusion, I wish at the outset to define some of the terms I may use, and mark out what I understand to be the meaning of the named topics.

ANESTHETICS, GENERAL AND LOCAL.

Anesthetic, adjective of anesthesia. *Aesthesia* signifies perception sensibility. The coupling of the word *aesthesia* with the prefix *an* makes it mean literally, want of perception or sensibility; same as *sthenia*, strength; *asthenia*, want of strength; *hemia*, blood; *anemia*, lacking blood. Dr. Oliver Wendell Holmes gives anesthesia as the state or condition: a want of perception or sensibility, referring particularly to state or condition following etherization. Dunglison defines it, privation or sensation, especially that of touch; paralysis of sensibility, and gives it as general or local. He instances deafness (anesthesia acustica), anesthesia bulbar from injury of the pons varolii or medulla oblongata, anesthesia linguæ, loss of sense of taste, and twelve or thirteen others. I shall use the term in its more restricted sense, as a privation of sensation or sensibility caused by administration of some drug.

Analgesia, analgia; from *algos*, pain, the prefix *an* and *algia*, insensibility to pain, but not to impressions of touch or contact; for example, morphine in full dosage may act as an analgesic.

Obtundent, from *obtundo*, to beat against and blunt the edge; whatever partially or wholly benumbs the nerves of sensation, blunts the anguish of suffering, may be said to be obtundent.

With these definitions as given, kept in view, I think my meaning will be plain while treating of

ANESTHETICS, GENERAL AND LOCAL.

Undoubtedly the committee who named my subject had in view those agencies that are popularly supposed to have such powers rather than anesthesias, analgesics or obtundents, such as pressure on nerve trunks, bulbs, ganglia or peripheria. Complete anesthesias may result to a part of the body from accidental or intentional severance of, or pressure upon, a nerve. Of these I shall not speak, nor shall I follow the custom established by writers on anesthetics, that a history of the one named must be given. I shall assume that the members of this convention will perfectly understand me when I name chloroform, or cocaine, or ether, or gaseous oxide of nitrogen, and do not need, and would not be enlightened, by history or technical description, however extended or accurate. But you, in common with myself, are keenly alive to the necessities of more and more accurate knowledge of the *when* to use, of *the* anesthetic to *select*, *how* to administer the one chosen, and *how* the one selected operates; we are anxious to definitely and wisely settle the questions of success in the use of and the danger attending any or all. Whether, if there is danger, it is inherent in the drug or in the preparation and exhibition. Of all these and more I have been repeatedly and often reminded since I had the honor to give before your society some views in a paper entitled "Things Old, New and Useful in the Operating-Room." Concerning these questions I have received letters from all points of the compass, from near and far; more than I could devote time to answer, but not one asking of the history or technical formulas or mode of manufacture.

Taking a hint from this indication, I will consider only the practical, striving to answer some of the questions above given.

1. What demand is there for anesthetics, general or local, in the practice of our profession? I answer, the same that calls for their use in the practice of medicine and general surgery. Not alone where there is odontalgia or neuralgias, multiform as they are, but in general, when pain is encountered or to be induced, that is not to be borne without shock or diminution of vital force on the part of the sufferer. And so long as patients and patrons of the dental offices keep up the cry that rings in your ears and in mine of "Dentistry is cruel," "The persistent cruelty of dentists borders on brutishness," "Dental offices are inquisitorial places of torture," "I dread to enter a dental office," "I like you socially and as a friend, but I hate your profession," "Why did you, who are kind-hearted and humane, engage in so cruel a calling?" and so on and on, *ad infinitum*. So long as we hear these things there is demand for them.

So general is the charge, so universal the complaint, that little children who do not know the meaning of the word "dentist" have to be dragged across our thresholds, and with quivering lips, pallid with fear, breaths tremulant with dread, beseeching eyes often filled with tears, beg for mercy before they reach the chair. You know that what I say is true; you hear it in society, at the club, at weddings and funerals; we are caricatured in theatres, written about in the papers, valentined in February. It is the same cry on the street, in the shop, everywhere. It is our shame and disgrace. A long way are we behind the medical and surgical profession in pain avoidance. I verily believe that in the offices of the 26,000 and more dentists of the United States, there is more suffering, more agony deliberately caused daily than ever was borne in all the inquisitions of the world in a month.

What pain is severer during the time of its duration than that endured in the extraction of a tooth? What pang more unendurable than that caused by the thrust of steel on an inflamed pulp? What will send shivers down one's spine faster and colder than burring supersensitive dentine? What will bring profanity to the lips of real gentlemen and tears to the eyes and down the cheeks of women sooner than the grind of corundum or carborundum wheels across the cusps of teeth in preparation for crowns? Who likes the prod of the broach against the ultimate end of a root's pulp after arsenious paste has not devitalized it through its entire length? On one occasion I was in the office of a friend while such prodding was going on. The patient, a stalwart man, groaned, twisted and perspired till my friend innocently inquired, "Does it hurt?" Instantly the patient replied, "Yes, damn you, it does hurt!" and in a towering rage left the chair and the office. There is not a thoughtful man in this presence but knows that thousands of teeth are sacrificed daily in "painless extracting rooms" and "painless dental parlors," and tens of thousands of dollars kept from the pockets of conscientious, reputable dentists, because of the dread of such suffering as I have described, now supposed to be necessarily incident to repairing and preserving the teeth. Hence I answer in repetition and (particularizing) in all oral surgery, whenever the soft tissues of the mouth are to be wounded, the gingivæ pressed upon or disturbed painfully, in all extractions of teeth, deciduous or permanent, when fright to child or adult attends, and in all burring, cutting or grinding of sensitive dentine, in all operations upon or painfully contiguous to sensitive pulps, if absence of pain is desired by the patient, anesthetics, general or local, to be used in full general anesthetization or in local anesthesias as the several cases require, should be in the hands of every operative dentist for daily, careful and intelligent use.

### THE ANESTHETIC TO SELECT.

In all grave operations requiring time and repose of the patient, such as excison of the inferior maxilla, osseous removals of the superior maxilla, for the removal of tumors of any considerable size, whether benign or carcinomatous, excison, stretching or extirpation of inferior dental nerve, most operations for cleft palate, removal of adenoid growths from posterior nares, and others not necessary to name, a general anesthetic should be given where local anesthesia is not adequate.

### WHAT ANESTHETIC FOR LOCAL USE?

Fortunately for your endurance in listening the enumeration can be brief. Passing cold, heat, nerve pressure and that residing in some of the essential oils, I shall only name one, erythroxylon coca and derivatives. To my mind, all others have been tried and found wanting. Till further search shall reveal agencies as efficient, it were a waste of time to speak of any other. I do not hesitate to place tropa cocaine at the van; it is the prince royal of the coca line. (Exhibit.) The next one in importance is cocaine hydrochlorate or cocaine muriate. Unfortunately, the price of tropa cocaine is so high that it will not come into general use at present. I have been unable to procure it for less than thirty cents a grain, still, wherever a local anesthesia was needed, if I had to rely upon hypodermic use, I should pay the price, provided also that the muriate could not be as safely and beneficially employed. It is difficult for me to draw a just comparison between the two preparations, for I have used the tropa cocaine but sparingly.

For the benefit of those who must rely on hypodermic use, I will say that a 2 per cent. solution of tropa cocaine is of ample strength; and I believe it is also safe to the amount of 4 to 6 drops injected, unless a blood-vessel of some size is pierced or a nerve of appreciable size is severed. In the former case, the drug might flow directly to the heart and cause collapse, perhaps death; in the latter case, paralysis of the supplied parts. Several cases have come under my notice of paralysis; one of the tongue, continuing some months; another of the left side of the face, lasting weeks; a third, a complete paralysis of the left arm, which lasted several weeks; all from injections by operators of so-called "painless dental parlors."

These cases do not argue, however, against the skillful use of cocaine. None but a coward will hesitate to skillfully, carefully use any beneficient agent because with them some have ignorantly or carelessly caused death or paralysis. It is almost, if not quite, axiomatic that an agent potent for good is also strong for evil.

The cocaines are no exception, but I am addressing educated, learned men. You have traced the course of nerve, artery and vein and know how to avoid them in the thrusts of your needle point.

Theory suggests and experience demonstrates that cocaine hydrochlorate can be safely injected, not only for tooth-extraction, but for many surgical operations, and render them absolutely painless. For local anesthesia, the following formula is commended in theory and practice as efficacious and safe, no accidents or deaths having followed its oft-repeated use.

| | | |
|---|---|---|
| R. | Sulph. atrop, - | gr. ½. |
| | Sulph. strophanthin - | gr. 1-5. |
| | Acid carbolic (crystals), | gr. v. |
| | Hydrochlor. cocaine - | grs. xx. |
| | Glycerin (pure) - | fl. dr. ss. |
| | Aq. pur. ad., - - | oz. ii. |

M. Sig.—Four to six drops hypodermically. I have given this formula before, and during the years that have intervened, it has been in constant use, not in the extraction of teeth and roots only, but for minor surgery by one of Chicago's noted surgeons, Dr. Oscar J. Price, and by many dentists, and so far without casualty or collapse; nor is there subsequent sloughing or inflammations.

I commend it without hesitation as efficacious and safe. While I do this, I wish to be as emphatic in my caution against the use of cocaine muriate *alone*, no matter if the solution is as low as 1 or 2 per cent.

In my own practice, alarming symptoms have occurred following a couple of drops of 4 per cent. solution in the eyes, one drop in each, preparatory to operating for ophthalmoncus. Nausea, facial pallor, general nervous tremors have supervened the application of a similar strength solution to the anterior nares for catarrhal congestion, as in sudden colds and hay fevers, and so marked have been these disturbances that I have entirely abandoned the use of any solution of cocaine, unless guarded by some toxical antagonist for that purpose, I recommend strophanthin sulphas, and atropin sulphas. A favorite formula is:

| | | | |
|---|---|---|---|
| R. | Muriate cocaine, | - | grs. xxxviii. |
| | Sulph. atropin | - - | grs. ¼. |
| | Sulp. strophanthin, | - | grs. 1-5. |
| | Beta naphthol, | - - | gr. 1. |
| | Aqua dist. ad., | - | fl. dr. ii. |

M. Sig.—For hypodermic or topical application. The cocaine should be in clean, well-formed crystals, and not in fine powder. (Exhibit.)

Do any of you say that so small an amount of strophanthin or atropin cannot avail in two ounces of the mixture? To such I reply, test these drugs upon yourselves, as I have done on myself. Place 1-60 of a grain of strophanthin well back on your tongue, wait a little, then take a mirror in hand, and if you are a good colorist, you may define the shade of red or purple that overspreads your face; then strive to detect in yourself some symptoms of syncope. Again, lay 1-40 of a grain of atropin on your tongue, and soon a dryness will pervade your throat and mouth, so that you can not spit for a half day. If you then look well to your eyes, you will see that it is a mydriatic of much potency. Better than theory of objection or approval, experience has shown that, combined with cocaine, they add potency to it, while divesting it of its toxic terrors. There are in some of the essential oils anesthetic properties, and in some excipients, as lanolin and glycerin, conveying tramways for cocaine, which act as adjuvants and are valuable aids to the main drug. So well convinced am I of it from personal experience, that I shall risk your merriment while I here repeat the formula I gave on a former occasion. For the purposes of experiment in reference mainly to this paper, I have tried to supplement the "pigment" by other combinations, simpler and yet as efficient. The result has been a return to the original formula with slight modification, increasing the amount of strophanthin and atropina. For nearly eight years I have proven its value and tested it safely.

### COMPOUND COCAINE PIGMENT.

| | | |
|---|---|---|
| R. | Atropin sulph., - - | gr. i. |
| | Strophan. sulph., - | gr. i. |
| | Cocaine hydrochlor., - | dr. ii. |
| | Beta naphthol, - - | grs. x. |
| | Oil caryophil, - - | fl. dr. ii. |
| | Chloroform pur., - | fl. dr. ii. |
| | Glycerin to make, - - | fl. oz. i. |

M. Sig.—Use with applicator or hypodermically for local anesthesia.

So use cocaine wisely, cautiously, but fearlessly if wisely, remembering that the allectation of science has led its votaries to the discovery of few agents exceeding in value that which Nature has poured from her alembic into the frond of the erythroxylon coca.

### GENERAL ANESTHETICS.

This and other lands have furnished us with a larger number of agents possessing general anesthetic properties than is generally supposed. Time and space forbid the mention of but few, and the description of the effect and mode of administration of but two or three will be dwelt upon. At the head of the list as regards age and public esteem, I name alcohol. I need only to name it and add a few words as to its mode of administration for anesthesia before dismissing it.

To those timid as to the use of more vigorous agents it contains real value. Its administration should be preceded by a narcotic sedative, as the anesthetic stage is always preceded by one of excitement. Immediately following the administration of some hypnotic, as morphine or atropine, the administration of some form of alcoholic liquor should be commenced. If brandy or whisky is chosen, it is best given in teaspoonful doses, following a glass of water at first, at intervals of from three to five minutes. The intoxication may be carried to such an extent as the operator may desire.

By the exercise of self-control and a personal hypnotic command, an operator can extract a required number of teeth or perform a minor operation in surgery with very little pain to the patient.

The other general anesthetics I name are ether, pental, gaseous oxide of nitrogen and chloroform.

Ethyl bromide, perhaps, should be named. It is quite generally used, and of undoubted efficacy; but its administration is accompanied by cramp-like spasms and cyanotic congestions that to my mind render it of far less value and quite as dangerous as the others. The literature of ether is so voluminous that I feel justified in dismissing it with a few comments. Its disgusting odor, the tardiness of the narcosis, and the distressing subsequent effects effectually bar it for general use in dental offices. However, if used for full anesthesia, a purer and more highly concentrated preparation than is generally used should be sought; and when administered, the patient should not be condemned to breathe contaminated ether and vitiated air from the lungs into and from a tightly-fitting mask held closely over mouth and nose. A chance for a little pure air and an uncontaminated drug should be given the sufferer. (Exhibited mask and inhaler.)

### PENTAL.

This is a comparatively new anesthetic. In its present state and purity it was introduced to the surgical profession late in the year 1891.

It is a colorless liquid, of low specific gravity, insoluble in water, but miscible with alcohol, chloroform and ether. It is highly inflammable, but of stable composition; does not, like chloroform, decompose on exposure to light and air.

Hollander, Von Rognar, Von Merring, Weber and Mebes claim for it freedom from dangerous or unpleasant symptoms during nercosis or upon awakening.

Being a tri-methyl ethelyne, $C_5H_{10}$, and perhaps related closely in its anesthetic effects to what Dr. Richardson considers the safest of all (methylic ether), I think we may indulge large expectations for it. Since its introduction in (nearly) 1892, but a short time has elapsed in which to try any drug, any agent that shall seize the vital forces of life, volition, resistance muscular and neurotic, against which, sooner or later, we must perhaps write over against it "Wanting." It possesses one feature that commends itself to my judgment, and that is that consciousness is retained even when there is insensibility to pain; in that respect simulating what I term the analgic or obtundent influence of chloroform. It may be administered as is ether chloroform, from an inhaler like Young's or from a mask such as is generally used, or from a cone, but better, for dental purposes, from the mouth of a bottle, as I advise for chloroform. (Exhibit.)

### LAUGHING GAS.

Gaseous oxide of nitrogen, a substance so well-known yet little understood, capable of rapid imbibition, of great power, bland, easily inhaled, praised by many, feared by the majority, is worthy of more than a passing notice; but time and space will compel a briefer discussion than I would be otherwise tempted to give.

It is now generally supplied to the dental profession in a liquefied form, to be released into and given from rubber bags, or far better, into a gasometer of ample size, allowing the gas, when liberated from the cylinder, to stand over a supply of water, that it (the water) may withdraw from the gas any acidulated or other impurities. Thus treated and used in my hands, it has proven unvaryingly efficacious and assuringly safe. Contrary to all accepted teaching of school and magazine, I assert its exhibition should always be accompanied by a generous supply of atmospheric air, when thus exhibited, the sleep is profound, though a little tardier in coming on, and there is an entire absence of spasmodic convulsions and cyanotic congestion.

From an experience with it covering a period of more than thirty years, when thus purified by water accompanied by a sufficiency of air, I cease to feel the slightest fear in its exhibition; I consider it an indispensable necessity in a well-equipped office, and as an aside, I may be allowed to say that the full-fledged dentist should rarely extract teeth, but when such extraction is necessary, he should be prepared for, and should perform, the operation himself, thus inducing him to extract but few teeth and preventing the savagery too often practiced by those whose profession is tooth-extraction.

I think I should add that before its exhibition the patient should be sent to the water-closet, and under no circumstances should it be given to pregnant women.—*Western Dental Journal.*

[To be continued.]

